# BUB3, beyond the Simple Role of Partner

**DOI:** 10.3390/pharmaceutics14051084

**Published:** 2022-05-18

**Authors:** Patrícia M. A. Silva, Hassan Bousbaa

**Affiliations:** 1 UNIPRO—Oral Pathology and Rehabilitation Research Unit, University Institute of Health Sciences (IUCS), University Polytechnic Higher Education Cooperative (CESPU), Rua Central de Gandra, 4585-116 Gandra, Portugal; patricia.silva@cespu.pt; 2 TOXRUN—Toxicology Research Unit, University Institute of Health Sciences (IUCS), University Polytechnic Higher Education Cooperative (CESPU), Rua Central de Gandra, 4585-116 Gandra, Portugal; 3 Centro Interdisciplinar de Investigação Marinha e Ambiental (CIIMAR), Universidade do Porto, Terminal de Cruzeiros do Porto de Leixões, Av. General Norton de Matos s/n, 4450-208 Matosinhos, Portugal

**Keywords:** BUB3, spindle assembly checkpoint, mitosis, cancer, senescence, anticancer target

## Abstract

The BUB3 protein plays a key role in the activation of the spindle assembly checkpoint (SAC), a ubiquitous surveillance mechanism that ensures the fidelity of chromosome segregation in mitosis and, consequently, prevents chromosome mis-segregation and aneuploidy. Besides its role in SAC signaling, BUB3 regulates chromosome attachment to the spindle microtubules. It is also involved in telomere replication and maintenance. Deficiency of the BUB3 gene has been closely linked to premature aging. Upregulation of the BUB3 gene has been found in a variety of human cancers and is associated with poor prognoses. Here, we review the structure and functions of BUB3 in mitosis, its expression in cancer and association with survival prognoses, and its potential as an anticancer target.

## 1. Introduction

BUB3 belongs to the conserved budding uninhibited by benomyl (BUB) protein family, which is known to function in the spindle assembly checkpoint (SAC) pathway, from yeast to mammals [1,2]. The SAC monitors and ensures appropriate attachments of spindle microtubules to kinetochores, delaying anaphase onset until all chromosomes have been attached to the mitotic spindle in a bi-oriented manner, thereby preventing chromosome mis-segregation and aneuploidy, a driving force of tumorigenesis [1,2]. BUB3 is also involved in establishing kinetochore–microtubule attachment [3,4,5,6]. It recruits BUBR1 to kinetochores to form functional complexes which, together with MAD2 and CDC20, form the mitotic checkpoint complex (MCC), in charge of inhibiting the anaphase-promoting complex (APC) [7]. When all kinetochores are attached to microtubules and aligned at the metaphase plate, the MCC is dissociated, and no more MCC is formed, which releases the SAC to promote exit from mitosis. Besides its role in SAC signaling, BUB3 has been implicated in proper telomere replication and maintenance, as well as in the regulation of aging [8,9,10]. In addition, a number of studies have reported deregulated expression of the *BUB3* gene in human cancers; however, its role in carcinogenesis is still controversial [11,12,13]. Here, we reviewed BUB3′s functions in cell cycle progression, its roles in human cancers, and its potential as a target for cancer treatment.

## 2. The Structure of BUB3 and Its Functions in Mitosis

**Figure 1:**
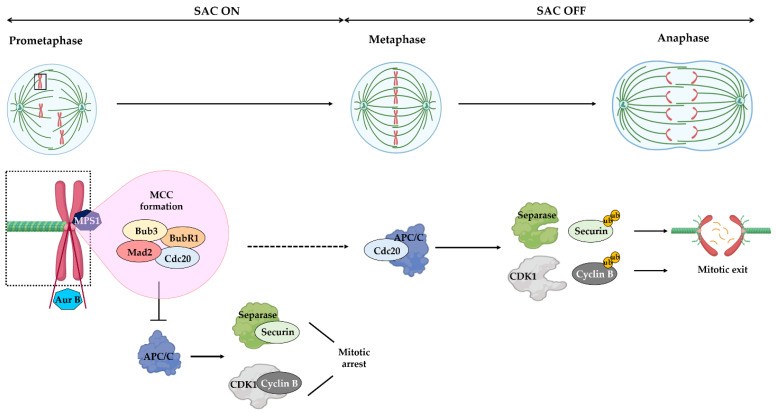
Spindle assembly checkpoint mechanism. In response to unattached or improperly attached kinetochores (prometaphase), the SAC is turned ON and promotes the assembly of the mitotic checkpoint complex (MCC), made of MAD2, BUB3, BUBR1 and CDC20. At these kinetochores, the SAC kinase MPS1 recruits BUB3, BUB1 and BUBR1. The MCC inhibits the activity of anaphase-promoting complex/cyclosome (APC/C), leading to the stabilization of separase/securin and CDK1/cyclin B complexes and, consequently, mitotic arrest. The Aurora B kinase (AUR B), associated with centromere heterochromatin, promotes proper kinetochore–microtubule attachments. Once all chromosomes are properly attached to spindle microtubules and are aligned at metaphase plate (metaphase), the SAC is turned OFF, through MCC disassembly, and, consequently, CDC20 can bind and activate the APC/C, resulting in ubiquitylation (ub) of cyclin B and securin mitotic subtracts. In turn, separase can cleave cohesins to promote sister chromatid separation (anaphase), while CDK1 inactivation promotes exit from mitosis. Reprinted from [23], MDPI 2021.

*BUB3* belongs to a series of genes (*BUB1*, *BUB2*, *BUB3*, *MAD1*, *MAD2*, *MAD3*, and *MPS1*) identified in a genetic screen in the budding yeast Saccharomyces cerevisiae that failed to arrest in response to spindle damage [14,15,16]. Mutants of these genes prematurely exit mitosis in the presence of microtubule-depolymerizing drugs, thereby accumulating severe mitotic errors. Functional orthologs of these genes were also identified in higher eukaryotes, including humans [17,18,19,20]. After nuclear envelope breakdown at the prophase to prometaphase transition, each of the two kinetochores formed at the centromeric regions of paired sister chromatids initiates attachment to spindle microtubules, with the goal of achieving bi-orientation with sister kinetochores oriented toward opposite poles of the spindle. However, in response to unattached kinetochores, the SAC is activated, through hierarchical recruitment of SAC proteins, including MAD1, MAD2, BUB3, BUBR1, and CENP-E, at the unattached kinetochores [21,22]. Consequently, MAD2, BUBR1, and BUB3 form a complex with CDC20, known as the mitotic checkpoint complex (MCC) [7] (Figure 1). This way, CDC20 is sequestered and prevented from activating the anaphase-promoting complex/cyclosome (APC/C), which is a ubiquitin E3 ligase that promotes proteolysis of securin and cyclin B and subsequent mitotic exit. Proper bipolar attachments of all chromosomes to the mitotic spindle leads to SAC inactivation, allowing CDC20 release and APC/C activation, which promotes anaphase entry.

**Figure 2:**
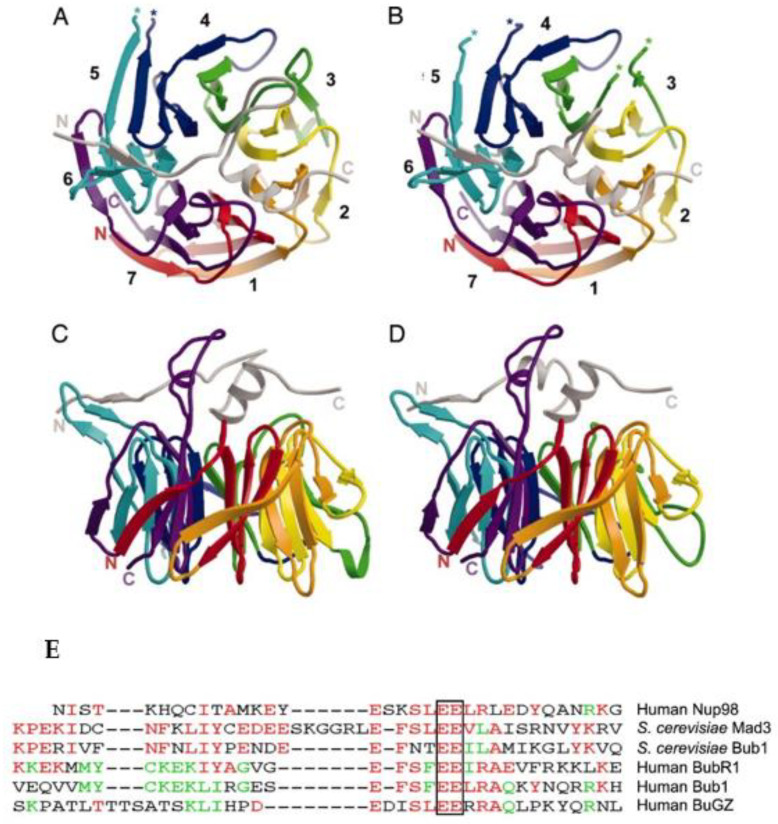
Top overview of Bub3 bound to GLEBS motif determined in yeast. (**A**) Top view of Bub3 bound to the GLEBS motif from Mad3 (BUBR1 in higher eukaryotes). The GLEBS peptide is colored in gray and lies along the top face of the propeller. N and C termini are labeled, and breaks in main-chain density are denoted with asterisks (*). (**B**) Top view of Bub3 bound to GLEBS motif from Bub1. Overall, the structures are quite similar, except that the Bub1 GLEBS motif has a shorter loop between helices α1 and α2. (**C**,**D**) Side views of Bub3 bound to Mad3/BUBR1 (**C**) and Bub1 (**D**) GLEBS motifs. The three-stranded β-sheet that includes the DA loop between blades 5 and 6 of Bub3 projects leftward in these views. Numbers indicate blades. Reprinted with permission from [24]. Copyright 2021, National Academy of Sciences, U.S.A. (**E**) The conserved GLEBS motif in BUB1 and BUBR1 that binds BUB3; alignment of GLEBS motifs from hNUP98, scMad3, scBub1, hBUBR1, mBUB1, and hBuGZ; the completely conserved amino acids, EE, are boxed. Reprinted with permission from [37]. Copyright 2022, Elsevier.

BUB3 is a fundamental piece in SAC activation. At the molecular level, BUB3 is a WD40-repeat protein with a seven-blade β-propeller structure forming a symmetric circular wall around a central pore, giving it a donut-like shape [24,25,26] (Figure 2A–D). BUB3 binds to phosphorylated MELT repeats (Met-Glu-Leu-Thr(P)) on the outer kinetochore subunit Knl1 (also known as Spc7, BLINKIN, CASC5 in different organisms), resulting in the recruitment of its binding partners BUBR1 and BUB1 to kinetochores [27,28,29,30]. The BUB3–BUB1 and BUB3–BUBR1 interactions involve the BUB3 central pore region and the respective Gle2 binding sequence (GLEBS) motif of BUBR1/BUB1 (Figure 2E) [31]. Specifically, the interaction of BUB3 with the BUBR1 GLEBS motif is essential for the integrity of the human MCC, as mutations that disrupt this interaction interface result in SAC deficiency and chromosome instability in yeast and human cells [24,32]. Interestingly, specific disruption of endogenous BUBR1–BUB3 complexes in cancer cells phenocopies the effects observed in gene-targeting experiments [33]. Besides its role in SAC signaling, BUB3 promotes the establishment of correct kinetochore–microtubule (K-MT) attachments, in concert with BUB1 and BUBR1, probably through an antagonistic interaction between BUB3 and the motor protein dynein [5,6,34,35]. For this purpose, BuGZ, a GLEBS domain-containing and kinetochore-binding protein, interacts with and stabilizes BUB3 to facilitate its kinetochore targeting and function in K-MT attachment [36]. This interaction was disrupted when the highly conserved EE to AA of the BuGZ GLEBS motif was mutated (see the outlined EE in Figure 2E) [37].The specific contribution of each BUB protein in the regulation of K-MT attachments remains difficult to assign, given the dependency of BUB1 and BUBR1 on BUB3 for kinetochore localization [6]. We previously addressed the specific contribution of BUB3 to K-MT attachment in human cells, and compared it to that of BUB1 and BUBR1, using an RNA interference (RNAi) approach and high-resolution microscopy [5,6]. We found that BUB3-depleted cells exhibited microtubules running past the kinetochore pairs, similarly to BUB1-depleted cells, suggesting side-on binding to the walls of microtubules. In addition, chromosome alignment defects in the BUB3/BUB1 double depletion were worse than in BUB3 and BUB1 single RNAi, which is expected for proteins with specific and parallel functions in KT-MT regulation, suggesting a cooperative role. In contrast, BUBR1-depleted cells exhibited misaligned chromosomes, which, despite being detached from microtubules, have kinetochore pairs parallel to the spindle axis, suggesting that BubR1 is involved in K-MT stability rather than in microtubule binding. Thus, in our view, BUB1 and BUB3 seem to regulate the switching from lateral to end-on attachment, while BUBR1 is required for stabilization of K-MT attachments.

## 3. BUB3 in Aging

BUB3 shares extensive sequence homology with each of the four WD repeat motifs, and over the entire length of the RAE1 protein, indicative of functional similarity [31,38]. While BUB3 functions in the SAC pathway, RAE1 (also called Gle2 or mrnp41) is involved in mRNA export in interphase [38,39,40,41,42]. Binding to RAE1 is mediated by a GLEBS motif present in the nucleoporin Nup98 [41]. Strikingly, RAE1 also binds to the GLEBS motif of BUB1 [43]. The discovery that BUB3 also binds to the GLEBS motifs of the SAC proteins BUB1 and BUBR1 has led to the hypothesis that RAE1 might have a role as an SAC protein [43]. Homologous recombination-mediated mouse *Rae1* gene disruption showed that the loss of a single *Rae1* allele causes a SAC defect and chromosome mis-segregation. Besides the 34% identity and 52% similarity of the human RAE1 and BUB3, *Bub3* haploinsufficient cells exhibit a strikingly similar mitotic phenotype, suggesting that RAE1 and BUB3 are functionally analogous, namely, by playing a specific or perhaps a redundant role in BUB1 targeting to unattached kinetochores and subsequent SAC activation [26,44]. Interestingly, double *Rae1*/*Bub3* haploinsufficiency causes a much more severe chromosomal instability phenotype than single haploinsufficiencies, suggesting a cooperative role of RAE1 and BUB3 in regulating the SAC activities to prevent chromosomal mis-segregation [44]. Long-term phenotype analysis showed a reduced lifespan of mice harboring the combined *Bub3* and *Rae1* haploinsufficiency, with phenotypes associated with aging appearing early in double haploinsufficient mice, while mice with single *Bub3* or *Rae1* haploinsufficiency were viable and had a normal appearance [9,10,44]. Aneuploidy in single haploinsufficient *Bub3* or *Rae1* mice increased dramatically with age, and increased further in double *Bub3*/*Rae1* haploinsufficient mice [10,44,45]. Curiously, mice with single or combined disruption of *Bub3* and *Rae1* were not predisposed to spontaneous tumorigenesis. Instead, *Bub3*/*Rae1* haploinsufficiency caused early onset of cellular senescence, which was due to SAC weakening, rather than to aneuploidy itself. Since the age-associated phenotypes exhibited by haploinsufficient *Bub3*/*Rae1* mice also occur in very old wild-type mice, then *Rae1* and *Bub3* were proposed to accelerate the aging process. Molecularly, haploinsufficient *Bub3*/*Rae1* mice embryonic fibroblasts (MEFs) accumulate high levels of cellular senescence inductors, including p16, p19, p21, and p53, but, surprisingly, no major signs of apoptosis, suggesting that haploinsufficiency of *Bub3* and *Rae1* accelerates aging through induction of cellular senescence [9,10,44,45]. Significantly, and similarly to haploinsufficient *Bub3*/*Rae1* mice, hypomorphic *BubR1* mice develop several aging-associated phenotypes at a very young age, including cataracts, lordokyphosis, loss of subcutaneous fat, and impaired wound healing [46]. However, hypomorphic *BubR1* mice had a much earlier onset of aging phenotypes, with many more senescent cells, than haploinsufficient *Bub3*/*Rae1* mice, indicating that the rate of premature aging is correlated with the level of induction of senescence. Therefore, in addition to oncogenic transformation, accelerated aging seems to be another major biological manifestation of a weakened SAC [10,46]. What determines if it is oncogenic transformation or accelerated aging that will take place in a deficient SAC background is unknown. It might depend on the extent of SAC deficiency and/or SAC component depletion.

## 4. BUB3 in Cancer

Defects in SAC activity lead to chromosome mis-segregation, which is thought to be responsible, at least in part, for aneuploidy generation in human malignancies [1,47,48,49,50]. SAC deficiency is often associated with deregulated SAC genes [1,48,50]. We examined the expression of *BUB3* in various human cancer types. To this end, *BUB3* gene expression and clinical data for 35 cancer types retrieved from the UALCAN data portal (http://ualcan.path.uab.edu/index.html, accessed on 24 December 2021) were analyzed [51]. *BUB3* transcript levels were compared between cancers and normal tissue in 18 cancer types; 17 cancer types were excluded from the analysis due to lack of normal samples. We found *BUB3* to be significantly overexpressed in cancers compared to normal tissue in 16 of the 18 cancer types analyzed (Table 1).

**Figure 3:**
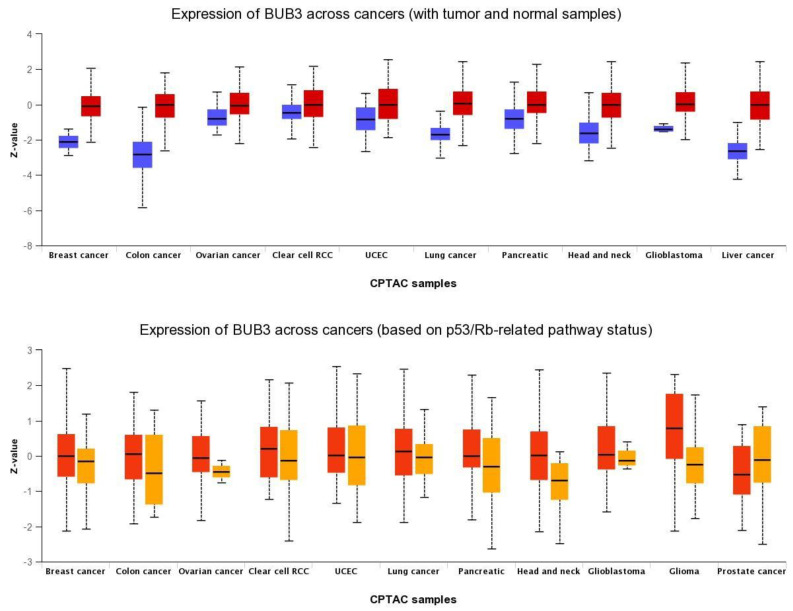
Pan-cancer view of expression of BUB3 protein across cancers. (**Upper panel**) Comparison between normal (blue) and primary tumors (red); (**lower panel**) Comparison between TP53-mutant (red) and TP53-non-mutant (orange) tumor samples. RCC: renal cell carcinoma; UCEC: Uterine corpus endometrial carcinoma; CPTAC: Clinical Proteomic Tumor Analysis Consortium. Data were retrieved from UALCAN portal (http://ualcan.path.uab.edu/index.html) on 24 December 2021.

BUB3 protein levels are also elevated in a wide variety of human cancers compared to normal tissue (Figure 3). We analyzed BUB3 protein levels in TP53-mutant cancers, as TP53-dependent SAC has been described [52,53,54]. TP53 is a transcription factor that acts as a tumor suppressor by inducing cell cycle arrest, cellular senescence, or apoptosis in response to cellular stresses, such as hypoxia, DNA and spindle damage [55]. *TP53* gene mutations are universal across cancer types, and this contributes to human cancers in different ways [56]. The TP53 pathway regulates the expression of a network of genes that are targeted to respond to a variety of intrinsic and extrinsic stress signals to ensure, among other things, accurate DNA replication, chromosome segregation, and cell division [57]. Interestingly, in most of the cancer types analyzed, BUB3 levels are significantly higher in *TP53*-mutant cancers than in *TP53*-wild-type cancers, suggesting that wild-type TP53 represses *BUB3* gene expression in physiological conditions, and that the TP53–BUB3 pathway may play an important role in carcinogenesis (Figure 3).

Previous studies have reported *BUB3* overexpression, at both RNA and protein levels, in a variety of human cancers compared with normal tissue. In most cancers, this upregulation was associated with poor prognoses. We reported that *BUB3* is upregulated and is associated with poor prognosis in oral squamous cell carcinoma [11]. The positive expression of cytoplasmic BUB3, together with that of cyclin B1 and the pituitary tumor-transforming gene 1, was significantly correlated with recurrence in prostate cancer [58]. *BUB3* was upregulated in 79% of gastric cancers, being a proliferation-dependent phenomenon in gastric cancer [13]. BUB3 levels were reported to be higher in sarcoma samples, and higher expression levels of BUB3 were associated with lower overall and disease-free survival in patients with sarcomas [59]. High expression of *BUB3* was associated with increased mortality in hepatocellular carcinoma [60]. In other studies, however, high protein expression of BUB3 in low-grade breast cancers was associated with longer overall survival, whereas lower expression resulted in poorer outcomes [61]. Upregulated *BUB3* was also reported in breast cancer samples [62]. Polymorphism in the *BUB3* gene was associated with the worst survival outcomes in early-stage non-small-cell lung cancer [63]. As with other SAC genes, epigenetic deregulation remains the most common alteration in the *BUB3* gene, while mutations at the sequence levels are rather rare and confer no increased cancer risk [1]. For instance, genetic variation in the *BUB3* gene did not affect familial breast cancer risk, and mutations in the *BUB3* gene were shown to be rare in bladder tumors and glioblastomas [64,65,66]. Overall, these studies confirm that the overexpression of the BUB3 gene and protein is a common feature of human cancers, being associated with poor prognosis.

Why is *BUB3* overexpressed in cancer cells? This question still remains unanswered. *BUB3* and other SAC genes are frequently overexpressed in cancer, and such overexpression is correlated with chromosomal instability [67]. It was reported that loss of major tumor suppressor pathways, such as RB and TP53 pathways, can lead to transcriptional upregulation of SAC genes through E2F promoters and, subsequently, to chromosome mis-segregation [68,69,70]. As suggested by our analysis (Figure 3), TP53 loss could also lead to *BUB3* upregulation, which should fuel chromosomal instability in cancer cells.

The role of *BUB3* in carcinogenesis is still unclear. Contradictory results have been reported from animal models. For instance, haploinsufficiency of *Bub3* causes an increase in chromosome instability in mice, but is not clearly associated with the frequency or the rate at which tumors appear in the animal [71]. Analysis of mice with reduced levels of *Bub3* has shown that mice have significant increases in the number of aneuploid fibroblasts, and are predisposed to chemical-induced lung tumorigenesis rather than spontaneous tumor development [44]. A tumor suppressor role has been suggested for Bub3 in a *Drosophila melanogaster* tumorigenesis model derived from knocking down SAC genes [72]. Indeed, when transplanted into adult flies, Bub3-deficient tumors displayed neoplastic growth, widespread chromosomal aneuploidy, and high proliferative potential. Overall, these studies reveal that aneuploidy induced by *BUB3* downregulation might not be sufficient to initiate tumorigenesis but might still facilitate it.

## 5. BUB3 as an Anticancer Therapeutic Target

For many years, the role of BUB3 has been reduced to the recruitment of its partners BUB1 and BUBR1 to unattached kinetochores. Probably for this reason, BUB3 has not been regarded as a potential anticancer target. Nevertheless, and as referred to above, BUB3 itself has a specific role in regulating kinetochore–microtubule attachments, and is involved in telomere replication maintenance and premature aging [5,8,10]. Importantly, the *BUB3* gene is upregulated in most cancers studied, which is generally associated with poor outcomes. Thus, BUB3 is not just a simple partner, and its targeting deserves attention. Today, there are no small molecules against BUB3, and the unique attempt to target BUB3 makes use of RNAi [11]. In this study, we have shown that RNAi-mediated inhibition of BUB3 was cytotoxic to OSCC cells and enhanced their chemosensitivity to cisplatin [11]. This antiproliferative activity of BUB3 inhibition against OSCC cells was recently confirmed by another group [73]. Very recently, we showed that inhibition of BUB3 compromises glioblastoma cell proliferation, mainly through senescence induction rather than by apoptosis, suggesting that premature senescence can be a viable approach to restrain cancer propagation [74]. Thus, oligonucleotide-based targeting of BUB3 could be a viable therapeutic approach. However, small-molecule inhibitors should be a better option due to RNAi security and stability issues. As BUB3 is a non-enzyme protein, and, thus, an “undruggable target”, the development of an anti-BUB3 drug may be a challenging task. To circumvent this, one should design small molecules that target protein–protein interactions to interfere with biological processes by modulating the formation of protein–protein complexes. In this sense, targeting the interaction of BUB3 with BUB1 and BUBR1 is an attractive option. This would prevent MCC formation, leading to SAC inactivation, which is expected to kill cancer cells as a consequence of massive chromosome mis-segregation. Strategies to mimic *Bub3*/*Rae1* haploinsufficiency in order to induce premature senescence of cancer cells should be explored. Indeed, cellular senescence has also been considered a suppressive mechanism of tumorigenesis, making therapy-induced senescence a plausible approach for cancer treatment, by irreversibly arresting the cell cycle [75].

## 6. Conclusions and Perspectives

Proteins of the SAC signaling pathway have been investigated as targets for the development of new antimitotic strategies for cancer treatment. Here, we have reviewed the role of BUB3 in mitosis and highlighted its potential as an anticancer therapeutic target. Besides its key role in K-MT attachment and SAC activation, BUB3 has a role in telomere replication and maintenance. Its upregulation is a common feature of human cancers, with higher expression in *TP53*-mutant cancers, and this could be a prognosis biomarker. Importantly, decreased levels of BUB3, either through genetic recombination or RNAi, accelerate aging through induction of senescence. Therefore, BUB3 could be a promising target for cancer treatment, namely, to increase sensitivity to radiotherapy and chemotherapy.

Some challenging issues need to be addressed to allow progress to clinical application. For instance, we need to know whether *BUB3* upregulation in cancer is a cause or just a consequence of the carcinogenesis process. Additionally, understanding how *TP53* regulates *BUB3* expression is important to explore the impact of BUB3 targeting, namely, for the treatment of *TP53*-mutant cancers. Elucidating the crosstalk between the SAC pathway and the senescence pathway is crucial for understanding the cellular mechanism of BUB3 targeting. These lines of study would provide additional insights into the role of BUB3 in carcinogenesis, which in turn might be useful for a rational drug design. Overall, we believe that BUB3 targeting could be a promising strategy for anticancer therapy that deserves to be explored.

## Figures and Tables

**Table 1:** Comparison of BUB3 expression between cancers and normal tissue.

Cancer Type	Fold Change ^1^	*p* Value ^2^
Bladder urothelial carcinoma	1.68	1.68 × 10^−12^
Breast invasive carcinoma	1.79	1.11 × 10^−16^
Cervical squamous cell carcinoma	2.24	3.72 × 10^−3^
Cholangiocarcinoma	3.96	5.06 × 10^−14^
Colon adenocarcinoma	1.51	1.62 × 10^−12^
Esophageal carcinoma	2.62	3.50 × 10^−8^
Glioblastoma multiforme	1.43	2.73 × 10^−1^
Head and neck squamous cell carcinoma	1.82	<1 × 10^−12^
Kidney chromophobe	0.38	<1 × 10^−12^
Liver hepatocellular carcinoma	2.16	<1 × 10^−12^
Lung adenocarcinoma	1.51	<1 × 10^−12^
Lung squamous cell carcinoma	1.86	<1 × 10^−12^
Prostate adenocarcinoma	1.11	6.02 × 10^−3^
Rectum adenocacinoma	1.43	1.62 × 10^−12^
Sarcoma	2.00	1.36 × 10^−1^
Stomach adenocarcinomna	2.17	1.62 × 10^−12^
Thyroid carcinoma	0.93	3.43 × 10^−9^
Uterine corpus endometrial carcinoma	1.43	<1 × 10^−12^

^1^ Mean *BUB3* expression in cancers/mean *BUB3* expression in normal tissue; ^2^ Student’s *t* test, *p* value < 0.05.

## Data Availability

Not applicable.
